# Growth rate, health and welfare in a dairy herd with natural suckling until 6–8 weeks of age: a case report

**DOI:** 10.1186/1751-0147-49-16

**Published:** 2007-06-23

**Authors:** Ann Margaret Grøndahl, Ellen Margrete Skancke, Cecilie Marie Mejdell, Johan Høgset Jansen

**Affiliations:** 1 Department of Companion Animal Clinical Sciences, Norwegian School of Veterinary Science, P.O. Box 8146 Dep., N-0033 Oslo, Norway; 2 National Veterinary Institute, P.O. Box 8156 Dep., N-0033 Oslo, Norway; 3 Department of Basal and Aquatic Medicine, Norwegian School of Veterinary Science, P.O. Box 8146 Dep., N-0033 Oslo, Norway

## Abstract

Over a period of two years, growth rate and health were measured for dairy calves allowed to suckle their mothers up to 6–8 weeks of age. Thirty-one calves were weighted weekly, and the mean daily growth rate was 1.2 ± 0.03 kg from birth up to 13 weeks of age. Illness in calves and young stock was not observed. In the cows, the mean incidences of ketosis, displaced abomasum, puerperal paresis, mastitis, teat injury and retained placenta were 0, 0, 8, 22, 1 and 1%, respectively, during a 6-year period. The mean daily gain of 56 growing bulls was 1.4 kg when slaughtered at 15 months of age, which is higher than the mean daily gain of 0.95 kg in the population. Probiotics, hormones and vaccines were not used, and antibiotics were only used for treating illness. The present study indicates many advantages and few problems when dairy calves are penned together with the cows and allowed natural feeding up to 6–8 weeks of age. This production system was easy to manage, preferred by the farmer, and may satisfy the public concern regarding the practice of immediate separation of cow and calf in commercial milk production.

## Background

In most dairy herds in North America and European countries, calves are separated from their mothers immediately or few hours after birth. During the first weeks of life, the calves are usually kept in individual pens, which prevent calves from suckling one another, minimize the spread of disease, and simplify feeding and disease detection. The calves are fed milk artificially, either by bucket or a teat feeding system. It is recommended to feed dairy calves milk twice daily an amount equivalent to 10% of their body weight, until weaning at about 6 weeks of age [1,2]. Normal oral and ingestive behaviour pattern is prohibited by separation of newborn calves from their mothers. The segregation eliminates the maternal care and the influence of adults on calf behaviour, and the individual penning restricts movements and social interference with other calves.

Calf mortality has been reported to range between 2–20% [3,4]. In one study, 48.7 – 72.5% of three groups of 120 calves were treated with antibiotics [5], and about 50% of farmers in North America have reported to use milk replacers containing antibiotics when raising dairy calves [6].

Artificial teat feeding allows dairy calves to express their natural suckling behaviour, and feeding milk *ad libitum *has been observed to improve both health and weight gain [7,8]. Separating calves and cows 4 days after birth has been associated with health advantages for the calf, compared to separation at birth [9]. Calves allowed to suckle their mother for 14 days gained weight at more than three times the rate of those separated during the first 24 hours [10]. However, the later separation was observed to increase the separation stress for both cow and calf.

The public concern to animal welfare is increasing. Already in 1981, the Commission of the European Communities, Brussels, arranged a seminar on "Welfare and husbandry of Calves" [11], with the aim of preparing more acceptable methods of calf husbandry. However, most dairy calves are still segregated from adults and artificially fed, although it is generally agreed that the environment offered an animal should allow the physiological functions within a normal range. In EU (Directive 91/630/EØF), farmers are not allowed to separate piglets from their mother earlier than 21 days of age. A newborn piglet and a newborn calf are comparable concerning development and immune status at birth.

Cattle are social animals that under natural conditions live in groups. The cow leaves the group before delivery and keeps her calf isolated the first days, and then introducing the calf to the group. The cows may start to leave their calves for longer periods from about 2 weeks of age, and calves are gradually weaned at approximately 6–8 months of age [10,12].

In a private Norwegian dairy herd, the calves were, for animal welfare reasons, allowed to stay together with their mothers for 6–8 weeks after birth. This management started in 1999, and has been performed since then. The present study was performed to get knowledge about weight gain, health and slaughter weights in this herd performing natural milk feeding.

## Case presentation

### Measurements

In 2003 and 2004, a total of 31 calves were weighed weekly from birth up to 13 weeks of age. Some registrations lack for certain calves, mainly because they were a few weeks old when they were included in the study. The weight used was Profilvågen WE 2108 weight (S.N. 2358, Max 6 000 kg/Min 1 kg).

The health and slaughter data in this herd was obtained from a central data bank described later. Because of the quote system and a local *fromage blanc *production on this farm, information concerning total milk production was not available, except that the quote on 70,000 litres milk per year was produced.

### Animals and Management

The herd included approximately 15 cows, which is the mean size of dairy herds in Norway. The animals were fed approximately 24% hay, 22% grass silage, 20% pasture and 34% grain during the year, in addition to mineral and vitamin concentrates. Probiotics, hormones, vaccines and navel cord dipping with disinfectant were not used. Antibiotics were prescribed by veterinarians only, for diagnosed bacterial disease, and were not used for preventive purposes. Mastitis treatments include all udder treatments, also during the non-lactating period. From May to October, the cows and suckling calves were on pasture, and from October to May hay and grass silage were given *ad libitum*. All animals were kept loose in an un-insulated barn, and the cows and suckling calves had access to outdoors areas throughout the year. Long barley straw was used as bedding, except for the feeding area which had concrete floor. The grain was given in the parlour. A short time before calving, the dams were moved into an individual pen for delivery, where they stayed with their calves for 2–3 days before returning to the herd. The suckling calves had access to a separate pen with a small opening not allowing the cows to enter. The calves were allowed to be with their mother for 6–8 weeks, and the cows were also milked in the parlour two times daily. The cows freely moved into the parlour leaving their calves behind with the rest of the herd.

After separation from cows and simultaneously weaning at 6–8 weeks of age, the calves were stabled in groups of 3–10 animals of approximately same age. At 6 months of age, heifers and bulls were segregated, and they were kept in separate pens with 3–10 animals in each pen, using straw as beddings. After weaning, the animals were given hay and grass silage *ad libitum*, and 0.5–2 kg grain per day increasing with age.

### Breeding and Herd Health Recording Systems in Norway

Norwegian Red (NRF), the most common dairy breed in Norway, is selected for milk production, low frequency of clinical mastitis, and for several other functional traits, including female fertility. The relative weight given to health and fertility in the total merit index, used for selection of sires, has increased gradually over the last 25 years. In 2003, 95.9% of the herds and 96.5% of the cows in Norway participated in the Norwegian Dairy Herd Recording System [13]. This data bank includes information from several sources, and individual health recording is integrated. In Norway, each case of veterinary treatment has been registered on an individual cow basis since 1975. Antibiotics and other drugs can be prescribed only by veterinarians in Norway, thus health recordings are viewed as reliable.

The yearly health situation for all individuals in the present herd was obtained from the Norwegian Dairy Health Herd Recording System. Information is given on the number of veterinary treatments of ketosis, puerperal paresis, mastitis including treatment during the non-lactating period, teat injury, retained placenta in addition to information about the average cell count in milk. The mean slaughter weight and slaughter age were also obtained for 56 bulls sent for slaughtering during the years 1999–2004.

### Observations

The mean (± s.d.) body weight and mean daily gain per week up to 13 weeks of age is presented in Table 1 [Additional file 1]. Illness and mortality in calves and young stock were not observed by the farmer or the veterinarian, nor was cross-suckling. One calf was stillborn. In Table 2 [Additional file 2], the number of cow treatments and mean cell count in milk are given per year for a 6-year period. The slaughter results of bulls during the same 6-year period are given in Table 3 [Additional file 3].

The farmer observed no problems, e.g. aggression, "lost" calves, cows stealing calves, when returning the cow and calf into the group 2–3 days after delivery. The calves stayed close to their mother the first two weeks. After two weeks of age, the calves usually stayed together during playing/running, sleeping and grassing. Play behaviour such as galloping, bucking, kicking and turnings were common, and often performed by several calves at the same time. The separation during milking seemed to be without distress for the cows and the calves. Milking problems were not observed, but during the suckling period, awareness of empty udders was needed to avoid milking these. The calves were abruptly separated from their mother at 6–8 weeks of age and the separation resulted in vocal responses by both the cow and the calf for 1–2 days. Calves at approximately the same age were separated simultaneously, and stabled in a separate pen, but able to see and hear the cows.

## Discussion

In the present study, the daily weight gain ranged between 0.9 – 1.3 kg per week up to 13 weeks of age, which is higher than the weight gain reported in other studies. According to Appleby et al. [7], calves offered milk *ad libitum *from a teat feeding system from birth until 2 weeks of age more than doubled their gained weight (0.85 kg/d) compared to bucket fed calves given milk twice daily at 5% of the body weight per meal (0.36 kg/d). Calves allowed to suckle their mother for 14 days gained weight at more than three times the rate (59.9 kg at 14 days of age) compared to those separated the first 24 hours (46.9 kg at 14 days of age), and the difference was maintained until at least day 28 [10]. In these two studies, milk offered *ad libitum *and natural suckling were superior to restricted feeding regarding weight gain. *Ad libitum *milk feeding systems were not observed to increase health problems by Appleby et al. [7] and Chua et al. [14]. A weight gain of 1.1 kg/d was observed the first 28 days of age in 28 dairy calves fed whole milk from an artificial teat [8].

The higher daily weight gain in the present study compared to previous studies may be explained by breed differences, the effect of natural stimulation of the oesophageal groove, a possible influence of natural ingestion on absorption of digestive nutrients and the adequate amount of milk. In addition, the effect of maternal contact may also influence on weight gain, as showed in a study by Krohn et al. [15], where calves kept with the cow for four days after birth but not allowed to suckle, gained weight twice the rate of gain in calves that had been separated at birth.

Resent studies with calves fed milk *ad libitum*, as well as the present study, indicate that feeding calves milk an amount approximately 10% of their body weight daily results in underfeeding, and the recommendations should be changed. According to Jasper and Weary [2], compensatory growth may not take place later in life despite high levels of intake if the opportunity for rapid weight gain in young calves is not met.

Calf illness was not observed by the farmer nor detected in the veterinary control, neither during the increased control during this study nor the other years with natural suckling. The natural feeding, which ensures the calves enough milk and the normal ingestive behaviour pattern, may explain the good health situation in this herd. Long wheat straw has been observed to be superior to granite fines, sand, rice hulls and wood shavings regarding scours days and days of treatment in calves [16], and straw as bedding material may therefore also partly explain the good health situation in the present herd. Diarrhea is reported in most studies involving calf feeding and health [2,4,7,14], and both diarrhea and respiratory diseases are well known problems in raising dairy calves. Calves fed milk *ad libitum *by artificial teat had fewer days with diarrhea compared to restricted, bucked-fed calves [7]. When separated 4 days after birth, calves showed fewer days of diarrhea compared to early separated calves [9]. However, many farmers associate *ad libitum *milk feeding with diarrhea, and this may be the main reason why *ad libitum *feeding is not widely used.

In the present herd, the incidence of calf death, ketosis, mastitis and teat injury was lower compared to the dairy population in Norway. According to the Norwegian Dairy Herd Recording System for 2003, the average incidence of calf death was 3%, the incidence of ketosis was 5%, puerperal paresis 6%, mastitis 28% and teat injury 3%.

It is difficult to explain why ketosis and dislocated abomasum were not diagnosed in this herd during a 6-year period. The animals were fed hay, grass, grain and concentrate, which is a simple diet that could promote development of ketosis and dislocated abomasum. The present study indicates that good animal welfare may influence on the development of these metabolic diseases. Placental retention was observed in one cow delivering a stillborn calf. According to Krohn et al. [17], several days of suckling by calves may have health benefits for the cow, such as reducing the incidence of mastitis and the incidence of placental retention. Restricted suckling systems with one or two suckling periods each day has been reported to reduce mastitis and to increase the total milk yield production [18].

The mean slaughter weight of NRF bulls in 2003 was 291 kg, with a mean slaughter age of 19.2 months [13]. In the present herd, the mean slaughter weight of 56 bulls was 301 kg, with a mean slaughter age of 15 months. This difference is difficult to explain, except that good welfare may influence on the weight gain. Illness in the bulls was not observed.

Early social contact facilitates the development of normal species-specific social behaviour [10,19]. Dairy cattle housed in groups need to learn certain social skills to successfully interact with group mates. Individually penned calves are shown to have low social rank within the group, and lower milk production level [15,17,20]. Thus the management in the present herd should be favourable concerning social skills and behaviour.

Flower and Weary [10] observed increased response as vocalization and movements to separation by both cows and calves when the calves were separated from their mother at 2 weeks rather than at one day of age. However, calves separated at 2 weeks of age gained weight at more than three times the rate of those separated at one day of age. In addition, delayed separation appeared to influence the development of calf social behaviour based on a behaviour test involving introduction to an unfamiliar calf. The authors concluded that the benefits in weight gain and social behaviour might outweigh the increase in response to separation. During the week of weaning, pair-housed calves were not observed to experience a stagnation of growth as did individually housed calves [14]. Although systematic behavioural registrations were not performed, the farmer reported that both cow and calf vocalized the first 1–2 days after separation. The farmer did not observe milk withdrawal by the cow after separation. The fact that the calves were well socialized to other calves and penned with these after weaning may have reduced the stress associated with separation from the mother.

In the present herd, the calves had sufficient space essential for play behaviour. Sufficient space increases the occurrence and quality of locomotory play, and is reported to have benefits for the developing animal [21,22]. To watch the natural suckling and communication between cow and calf, as well as the play behaviour of the calves increased the farmer's welfare at work, and, to his opinion, these benefits outweighed the stress response observed at separation, which was mainly vocalisation.

The present study indicates that calves suckling their mothers for 6–8 weeks of age have increased weight gain compared to calves fed artificially according to the recommendations. In addition, the good health of calves, growing bulls and cows indicate that natural milk feeding is important to the growth rate and health situation, as well as animal welfare.

In the present farm, there was no need for more labour when changing the management from conventional calf feeding system to natural suckling. The only rebuilding done, was to establish an opening between the calf pen and the cow area. In the author's opinion, natural suckling should be possible also for larger herds, and the greatest benefit is the easy calf feeding system securing good calf health. The farmer's effect may, however, influence on the degree of success using the natural suckling system.

## Conclusion

Weight gain and health in a dairy herd performing natural suckling up to 6–8 weeks of age revealed an mean weight gain of 1.2 kg/d for calves up to 13 weeks of age, and absence of illness in calves, young stock and growing bulls. The production system allows natural behaviour as suckling and play, and may satisfy the public concern regarding immediate separation of cow and calf in commercial milk production. Furthermore, the farmer found it easy to manage and preferable to conventional production.

## Competing interests

The author(s) declare that they have no competing interests.

## Authors' contributions

All authors have read and approved the final manuscript.

## Supplementary Material

Additional File 1 Growth rate, health and welfare in a dairy herd tables. Click here for file
