# Supplementary material for: Growth rate, health and welfare in a dairy herd with natural suckling until 6–8 weeks of age: a case report
Source: Acta Vet Scand. 2007 Jun 23;49(1):16. doi: 10.1186/1751-0147-49-16 (PMC1913533; doi:10.1186/1751-0147-49-16)
Supplement: Additional File 1 — Growth rate, health and welfare in a dairy herd tables. [file 1751-0147-49-16-S1.doc]

Table 1. The mean body weight (BW± SD) and mean daily gain (ADG± SD) per week of calves

from birth to 13 weeks of age

| Age  (weeks) | 0 (birth) | 1 | 2 | 3 | 4 | 5 | 6 | 7 | 8 | 9 | 10 | 11 | 12 | 13 |
| --- | --- | --- | --- | --- | --- | --- | --- | --- | --- | --- | --- | --- | --- | --- |
| BW, kg | 41  ±1.3 | 48  ±1.1 | 57  ±1.2 | 65  ±1.4 | 73  ±1.6 | 81  ±1.7 | 91  ±1.9 | 99  ±2.0 | 105  ±1.9 | 112  ±1.9 | 117  ±2.5 | 128  ±3.6 | 131  ±6.0 | 139  ±7.8 |
| No. of calves | 15 | 26 | 27 | 27 | 28 | 31 | 31 | 31 | 30 | 27 | 21 | 14 | 7 | 5 |
| ADG  kg/d |  | 1.1  ±0.15 | 1.3  ±0.07 | 1.1  ±0.1 | 1.2  ±0.05 | 1.2  ±0.06 | 1.3  ±0.07 | 1.1  ±0.05 | 1.0  ±0.08 | 1.0  ±0.07 | 1.0  ±0.09 | 1.2  ±0.10 | 0.9  ±0.12 | 1.2  ±0.12 |

Table 2. The number of yearly treatments in the cows, mean cell count in milk and number

of cows during the years 1999-2004, and the mean incidence of the diseases during this period

| Year | Ketosis and  abomasal  dislocation | Puerperal paresis | Mastitis | Teat injury | Retained  placenta | Mean cell count in milk, 1000/ml | Number of cows |
| --- | --- | --- | --- | --- | --- | --- | --- |
| 1999 | 0 | 0 | 4 | 0 | 0 | 121 | 13 |
| 2000 | 0 | 2 | 4 | 0 | 0 | 105 | 16 |
| 2001 | 0 | 0 | 6 | 1 | 1 | 134 | 14 |
| 2002 | 0 | 2 | 4 | 0 | 0 | 97 | 16 |
| 2003 | 0 | 2 | 0 | 0 | 0 | 126 | 14 |
| 2004 | 0 | 1 | 1 | 0 | * | 114 | 15 |
| Mean incidence | 0 % | 8 % | 22 % | 1.2 % | 1.5 % |  |  |

* this figure was not given in the Norwegian Dairy Health Herd Recording System report for 2004.

Table 3. The mean slaughter weight, slaughter age and daily gain of 56 bulls during the years 1999-2004

| Year | Number of bulls | Mean  slaughter weight, kg | Mean slaughter age, months | Mean  daily gain, kg/d |
| --- | --- | --- | --- | --- |
| 1999 | 14 | 299 | 16 | 1.24 |
| 2000 | 10 | 295 | 15 | 1.31 |
| 2001 | 10 | 255 | 12 | 1.42 |
| 2002 | 7 | 301 | 14 | 1.43 |
| 2003 | 7 | 344 | 16 | 1.43 |
| 2004 | 8 | 314 | 16 | 1.31 |
| Total number | 56 | 301 | 15 | 1.36 |

**Additional files**

Additional file 1

File format: DOC

Title: The mean body weight (BW± SD) and mean daily gain (ADG± SD) per week of calves from birth to 13 weeks of age.

Description: The table presents the mean body weight and mean daily gain per week of calves from birth to 13 weeks of age.

Additional file 2

File format: DOC

Title: The number of yearly treatments in the cows, mean cell count in milk and number

of cows during the years 1999-2004, and the mean incidence of the diseases during this period.

Description: The table presents the number of yearly treatments in the cows, mean cell count in milk and number

of cows during the years 1999-2004, and the mean incidence of the diseases during this period.

Additional file 3

File format: DOC

Title: The mean slaughter weight, slaughter age and daily gain of 56 bulls during the years 1999-2004.

Description: The table presents the mean slaughter weight, slaughter age and daily gain of 56 bulls during the years 1999-2004.
